# Effects of different scar types on flap viability in a skin flap model: an experimental study

**DOI:** 10.55730/1300-0144.5446

**Published:** 2022-05-12

**Authors:** Ceyhun UZUN, Can İlker DEMİR, Emrah Kağan YAŞAR, Mehdi ASADOV, Murat Şahin ALAGÖZ, Esra CİVRİZ, Çiğdem VURAL

**Affiliations:** 1 Department of Plastic, Reconstructive and Aesthetic Surgery, Derince Training and Research Hospital, Kocaeli, Turkey; 2 Department of Plastic, Reconstructive and Aesthetic Surgery, Faculty of Medicine, Kocaeli University, Kocaeli, Turkey; 3 Department of Pathology, Faculty of Medicine, Kocaeli University, Kocaeli, Turkey

**Keywords:** Rat, flap, McFarlane, scarred pedicle, traumatic tissue, midline scar

## Abstract

**Background/aim:**

The purpose of this study was to investigate the use of tissues with multiple traumas, scarred pedicles, and medial scarring as a flap.

**Materials and methods:**

Forty-eight rats were randomly divided into four equal groups. The modified McFarlane flap was chosen as the flap model. In Group 1 (control), a dorsal skin flap was elevated and then sutured back into original position. In the other groups, a two-phase procedure was used. In Group 2 (pedicle incision), scar tissue was created with a skin incision at the prospective pedicle site of the flap and then sutured to its original site. In Group 3 (preconditioning), multiple full-thickness traumas were performed along the entire flap body, and in Group 4 (middle incision) scar tissue was created with a skin incision at the prospective middle region of the flap. Then, after 45 days, dorsal flaps were raised in all rats and then sutured back into position. Seven days later, flap survival was evaluated through microangiography and histological evaluation of flap segments. Histopathological examination included assessment of the number of vessels, necrosis, infiltration with polymorphonuclear leukocytes, edema, fibrosis, inflammation, increase in fibroblast activity, and neovascularization.

**Results:**

The flap survival rates were 66.78% in Group 1, 68.05% in Group 2, 68.5% in Group 3, and 60.01% in Group 4. The flap survival rate was significantly lower in Group 4 (p < 0.05). There was no significant difference in flap survival between Groups 1, 2, and 3. On microangiographic examination, the vascular network extended more distally and was densest around the scar line in Group 2. Vascularization was poorest in Group 4. On histological examination, the number of vessels tended to be greatest in Groups 3 and 4 but this was not significantly different between groups (p < 0.05).

**Conclusion:**

The study findings showed that it may be possible to raise a flap from a previously mutilated site secondary to scar formation and multiple full-thickness traumas along the flap body. However, distal necrosis may occur in situations when the scar is positioned in the middle region of the prospective flap.

## 1. Introduction

Local flaps, which have an important role in reconstruction, allow the defect to be covered with similar color, thickness, and textured skin. These flaps are commonly used because they are technically simple to design, plan, and harvest. The most important problem associated with these flaps is unpredictable partial necrosis in the distal area [1–3]. It is believed that transferring traumatized tissue to another area will increase the risk of necrosis by decreasing the blood supply to the distal flap. For this reason, it is considered better practice to design flaps from healthy tissues rather than scarred areas [4]. However, hypoxia occurring in the scar area increases the release of angiogenic factors and stimulates neovascularization. This process, which forms the fundamental principle of the delay phenomenon, allows development of new vasculature from the scarred area in a two-stage procedure, such as the groin flap [5]. Therefore, it is of importance to investigate the effect of planning a flap with regard to preexisting traumatized tissue.

The pathophysiology of scar and wound healing are similar but also exhibit differences [6]. New vessel formation or angiogenesis proceed with wound healing. Some cytokines in wound healing, such as fibroblast growth factor (FGF), vascular endothelial growth factor (VEGF), angiotensin, platelet-derived growth factor (PDGF), macrophage growth factors (MCP), and transforming growth factor beta (TGF-b) are effective in angiogenesis, but FGF and VEGF are also effective in scar formation [6, 7]. Although the exact causes of hypertrophic scars remain unknown, delayed epithelialization, tension, increased dermal thickness, location in the body, or tissue loss increase the incidence of hypertrophic scarring [8]. The thickness of the scar affects the neovascularization of the scar and penetration of the vessels.

There are a few published experimental and clinical studies examining the effect of scarred tissue on flap viability. In these studies, it may be possible to raise a flap from a previously mutilated site, secondary to scar formation and pedicle injury [4] and it was stated that the viability of random patterned flaps removed from scarred tissue was similar to flaps planned from healthy skin [9]. Clinically, it has been reported that the use of flaps elevated from the abdomen with a preexisting scar in the midline and subsequent use in breast reconstruction increased the necrosis rates [10–12]. In addition, no vascular problem was observed in nipple reconstruction techniques in which the mastectomy scar was included [13]. Although these studies provide very valuable information, the reliability of standard flaps planned from tissues that have experienced recent trauma or in which there was a scar in the midline was not evaluated.

This study aimed to investigate the viability and safety of using standard flaps with experimentally inflicted defects. These were: 1) a midline scar; or 2) flap in which the pedicle was previously damaged; or 3) flap in which the body of the flap was traumatized through the full thickness. An additional aim was to investigate the safe and most effective period between trauma occurrence and harvesting of the flap that incorporated the experimentally inflicted defect.

## 2. Materials and methods

### 2.1. Animal management

This experimental study was performed in accordance with institutional guidelines. The protocol for the use of rats in this study was approved by Kocaeli University School of Medicine, Laboratory Animals Ethical Committee (project number: 2019/27). In the study, a total of 48 young adult female Sprague-Dawley rats, with an average age of 8 weeks and a weight of 250–350 g, were used. The animals were housed individually in suitable cages where the temperature was 22 ± 2 °C, humidity was in the range of 60%–70%, and a 12 h light and 12 h dark cycle was applied. No special diet was used. The operation site was cleaned with antiseptic betadine (10%) solution. Surgical technique was clean but not sterile. Rats were anesthetized by an intraperitoneal injection containing 80 mg/kg of ketamine hydrochloride and 10 mg/kg of xylazine. No additional dose was given to the rats during the operation. Two animals were excluded from the final study for the following reasons: in Group 2, one animal suffered from total necrosis of the flap; and one rat in Group 4 became too ill to be included.

### 2.2. Flap model

In the study, the rat dorsal skin flap, first described by McFarlane [14] and later modified by Khouri [15], was used. The vascular supply for the flap comes from the iliac branches of the bilateral iliolumbar artery. Khouri’s modification was chosen because the rate of necrosis in the distal region of the flap was more consistent compared to similar models. The flap was planned with a caudal base, 9 × 3 cm in size, placed in between the scapular and posterior iliac protrusions, with the base on the transverse line passing through the hip joints.

### 2.3. Surgical procedure

The study took 52 days from start to finish. The 48 rats were randomly assigned to four equally sized groups. The study schedule is summarized in Table 1.

#### Group 1: control group (C)

**Figure 1:**
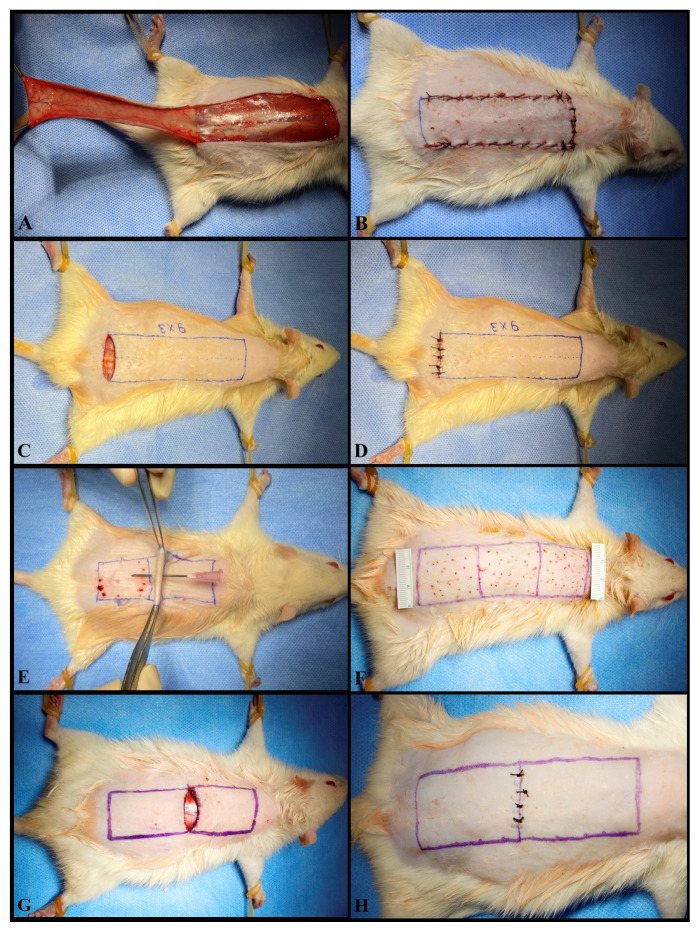
Procedure applied in Group 1 (Controls). The random pattern, inferior based dorsal skin flap was raised (A). It was then sutured back into the original place (B). The procedure used in the first session in Group 2 (pedicle incision). A full-thickness incision including the panniculus carnosus muscle was made on the flap pedicle between both iliac spurs (C). The skin incision was approximated (D). The procedure used in the first session in the Group 3 (preconditioning). The flap was divided into three equal parts (distal, middle, and proximal). The flap was lifted from its lateral borders using forceps and full-thickness holes were opened with an 18 G injector tip (E). A total of 72 holes were made (F). The procedure used in the first session in Group 4 (Middle Incision). A full-thickness transverse incision was made on the midline of the flap, including the panniculus carnosus muscle (G). The skin incision was approximated (H).

Firstly, the lateral borders of the flap were incised. After the skin and panniculus carnosus muscle were included in the flap and made bipediculated in the avascular plane over the muscle fascia. Then the distal border was incised and the flap was raised from the floor and then sutured back to its original place (Figures 1A and 1B).

#### Group 2: pedicle incision group (PI)

A two-step procedure was used. In the first stage, a 3-cm transverse incision was made through the skin and panniculus carnosus between the bilateral iliac protrusions. Direct pressure was applied to the wound until hemostasis was maintained. Wound edges were primarily sutured (Figures 1C and 1D). In the second stage, 45 days after the first procedure, a dorsal skin flap, which included the scar from the first procedure, was raised in all rats as an inferiorly based and scar-pedicled flap. Then flaps were sutured back into their original places.

#### Group 3: preconditioning group (PREC)

A two-step procedure was used. In the first stage, after the dorsal skin flap was planned, it was divided into three equal parts - distal, middle and proximal. Each region was lifted from opposite sides with forceps, and 72 holes were made in each flap using an 18 G pink thick needle (Figures 1E and 1F). In the second stage, again 45 days after the first procedure, a dorsal skin flap was raised in all rats as an inferiorly based flap. Flaps were again sutured back into their original places.

#### Group 4: middle incision group (MI)

A two-step procedure was used once more. In the first stage, a transverse, 3-cm, full-thickness incision was made in the middle of the proposed flap. Direct pressure was applied to the wound until hemostasis was maintained. The wound edges were primarily sutured (Figures 1G and 1H). In the second stage, again 45 days after the first procedure, a dorsal skin flap was raised in all rats as an inferiorly based flap. Flaps were again sutured back into their original places.

All of the rats were housed for 7 more days after the completion of the experimental procedures. Following this, the rats were sacrificed by high dose anesthetic medication and their dorsal skin was harvested for angiographic and histological examination.

### 2.4. Flap surface area measurements

All animals were photographed with a digital camera (Canon EOS 700D; Canon, Inc., Tokyo, Japan) under anesthesia with a ruler placed next to the object. The photographs were transferred to Adobe Reader XI (Adobe Systems®, Inc., San Jose, CA, USA) program and the distances were calibrated with the precision of one percent of a millimeter with the help of the ruler. The total area, healthy area, and necrotic area of each flap was measured in square millimeters and calculated as a percentage of the total.

### 2.5. Histopathological evaluation

Seven days after the first stage in the control group and 7 days after the second stage in the other groups, samples with a width of 1 cm (9 × 1 cm) were taken from the flaps. The samples were stored in 10% formalin for 24 h. After fixation, tissue samples were embedded in paraffin blocks and sections of 5 μm thickness were cut and stained with hematoxylin-eosin (H&E). The samples were examined and photographed with a Nikon E600 (Nicon Inc., Melville, USA) light microscope.

Polymorphonuclear leukocyte (PMNL) density, fibrosis, edema, findings indicative of inflammation, fibroblast activity, and neovascularization were evaluated over the whole area of the flap and scored between 0 and 4 (0 = absence, 1 = mild, 2 = moderate, 3 = severe, 4 = very severe). The number of vessels in an area of 1 mm^2^ in the healthy skin, 1 cm proximal to the necrosis border was calculated using the light microscope (Nicon E600) (×40) by the same pathologist blinded to the group of specimens.

### 2.6. Microangiography

Microangiography was performed on four randomly selected rats from each group; 7 days later, flaps were raised and sutured back into their original places. Rats were placed in a supine position under anesthesia. A vertical incision was made on the anterior chest wall and the intrathoracic space opened. While the rat was alive, the left ventricle was cannulated from the apex of the heart with a 20G intracet. Then 50 g of pure barium sulfate (30–35 cc volume), 5 g of gelatin, and 65 cc of saline prepared to a total volume of 100 cc was heated slowly on a low temperature heater, with stirring and then homogenized. The solution was introduced into the animal’s circulation via the left ventricle without cooling over a period of 10 min. As the solution enters circulation slowly, the rat died. After keeping the rat in the refrigerator at +2 degrees for 4 h, the flaps were removed and laid on a flat plate. The tissue was transferred to the image facility using a cold chain. The samples were all imaged using a mammography device (Fujifilm Amulet Innovality, Tokyo, Japan) and were then transferred to the computer.

### 2.7. Statistical evaluation

All statistical analyses were performed using IBM SPSS for Windows, version 20.0 (SPSS, Chicago, IL, USA). The Shapiro–Wilk test was used to assess the assumption of normality. Normally distributed continuous variables are presented as mean ± standard deviation while nonparametric data are presented as median (25th–75th percentile). Comparisons of continuous variables between groups were performed using one-way ANOVA or Kruskal–Wallis test, as appropriate. Tukey, Dunn, and Dunnett’s tests were used for multiple comparisons. All statistical analyses were carried out with 5% significance and a two-sided p-value < 0.05 was considered statistically significant. Two rats were excluded from the study. Therefore, we conducted a post hoc power analysis to determine the statistical effects of the rats excluded from the study. Post hoc power analysis was conducted based on the primary outcome “percentage of live area” using G*Power version 3.1.9.4. Test family was selected as” F tests”, the statistical test was selected as “ANOVA: Fixed effects, omnibus, one-way” and α was taken as 0.05 in the power analysis and power was calculated as 88%. This high statistical power indicates that the test results have a high likelihood of remaining valid.

## 3. Results

### 3.1. Flap survival

**Figure 2:**
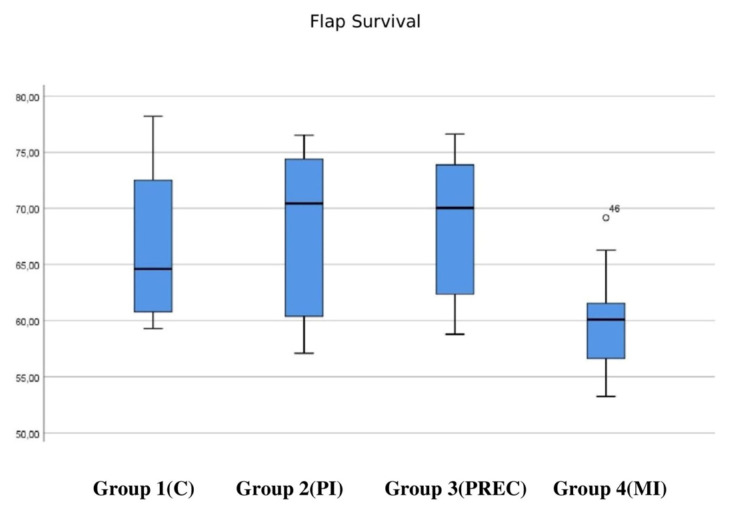
Graphical representation of the mean percentage of flap survival in Groups 1, 2, 3, and 4. Flap viability was highest in Group 3 and lowest in Group 4. There was no significant difference between the viability rate in Groups 2 and 3 and the control group. The viability rate in Group 4 was significantly poorer than the control group (p < 0.05).

**Figure 3:**
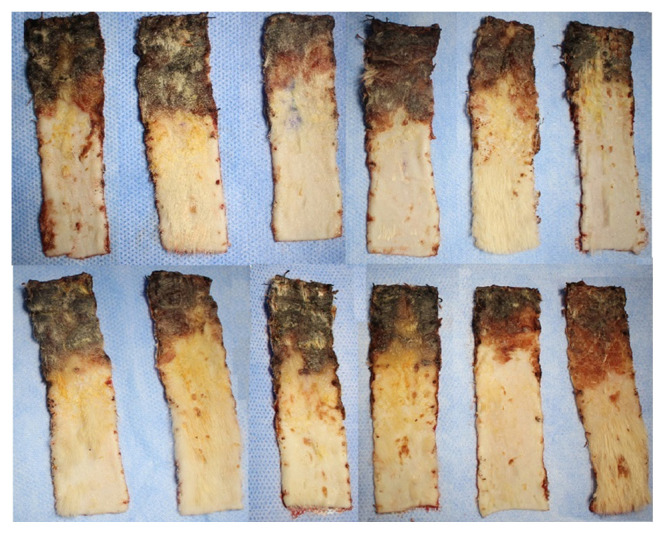
Group 1 (C) macroscopic images of flaps on the 7th day showing survived and necrosed areas.

**Figure 4:**
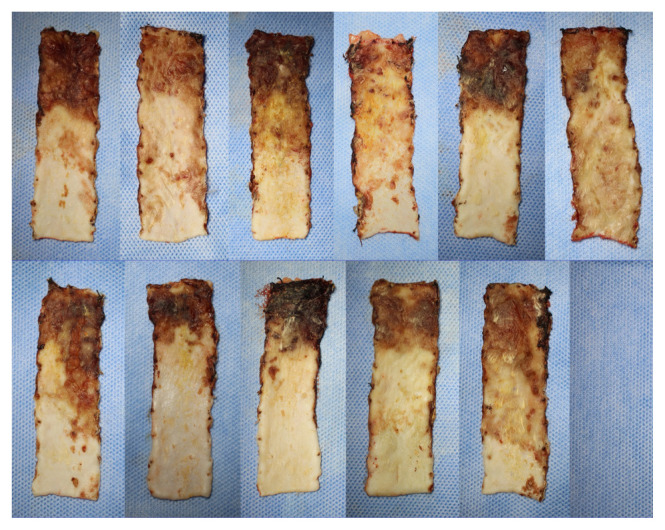
Group 2 (P) macroscopic images of flaps on the 7th day showing survived and necrosed areas.

**Figure 5:**
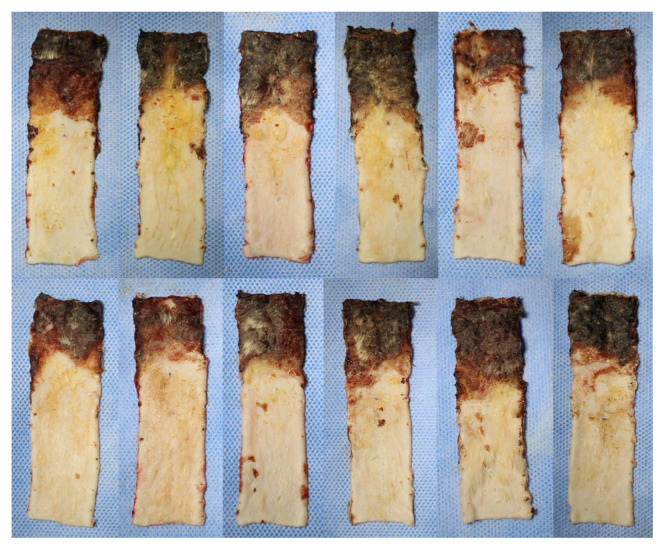
Group 3 (PREC) macroscopic images of flaps on the 7th day showing survived and necrosed areas.

**Figure 6:**
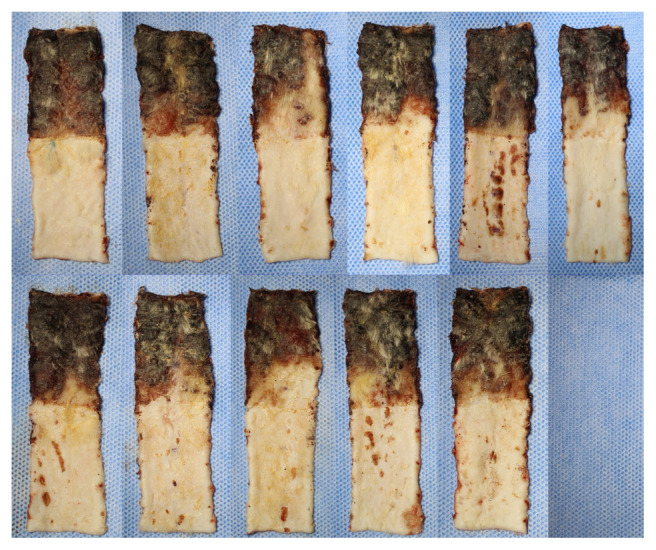
Group 4 (MI) macroscopic images of flaps on the 7th day showing survived and necrosed areas.

In order to minimize the effects of contraction of the flaps, flap survival rates were calculated by remeasuring the total flap area together with the survival flap area on the 7th day after flaps were raised. The flap survival rates were 66.78% in Group 1, 68.05% in Group 2, 68.5% in Group 3, and 60.01% in Group 4. Flap survival percentages were statistically different between the four groups (p = 0.011) (Table 2). There was no statistically significant difference in flap survival rate in Groups 1, 2, and 3 (p > 0.05) (Figure 2). In paired comparisons, the survival rate in Group 4 was significantly less than in all the other groups (Table 2). Macroscopically, in Groups 1, 2, and 3 the area of tissue that survived or necrosed were similar (Figures 3–5) but it was observed that viability was minimal in the distal region of the incision in Group 4 (Figure 6). An additional finding in this group was the absence of circulation in some subjects on microangiographic examination.

### 3.2. Microangiography

**Figure 7:**
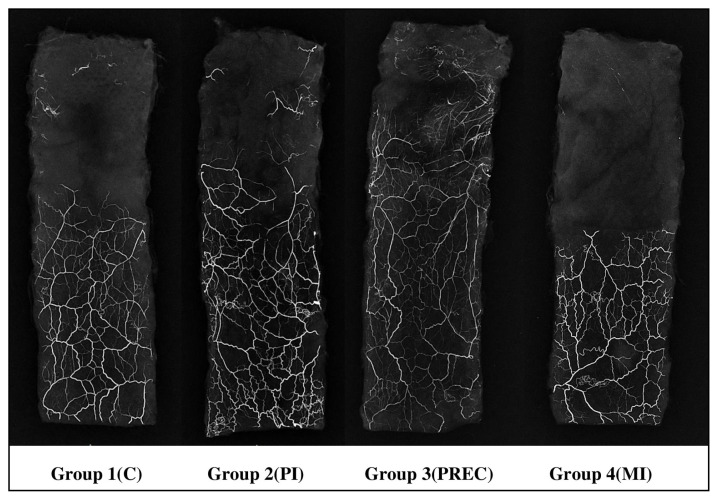
Skin flap microangiography in Groups 1, 2, 3, and 4. Group 3 had the densest distal vascular network. In Group 4, there was no capillary network in the distal half of the flap. In Group 2, the capillary network around the pedicle was the densest.

Microangiography was performed in four animals randomly selected from each group. Animals from the control group were accepted as the standard for the vascular network. It was observed that the vascular network progressed more distally in Group 2 compared to the control group and the vascular density around the scar line was also greatest in this group. Vascularization increased most markedly in Group 3 in the flap body and the capillary network was the most extensive in the distal portion of the flap. Vascularization was poorest in Group 4, so that no contrast material was observed distal to the scar in any of the four animals examined from this group (Figure 7).

### 3.3. Histological examination

When the number of vessels in the critical zone in an area of 1 mm^2^ was compared, the number of vessels tended to be higher in Groups 3 and 4, but this was not significantly different from the other groups (p = 0.058) (Table 3). When PMNL density, increased fibroblast activity and degree of fibrosis, edema, and inflammation, and neovascularization were evaluated, there was no significant difference between the four groups (Table 4).

## 4. Discussion

It is common that previous scars may be present near a defect that requires reconstruction, especially in high-energy traumas. As these scars may be associated with suboptimal vascular nutrition, surgeons often avoid the use of these tissues as flaps and prefer alternative options, such as distant flaps using healthy unscarred tissues [16]. The use of scarred, pedicled flaps, or a flap planned with a midline scar present or a fully body traumatized flap is controversial. For example, if a patient with a defect in the lower extremity has scars due to previous operations and traumas due to the use of an external fixator, a free flap may be preferred, considering that the use of local tissues will increase the risk of necrosis. These surgeries are complex procedures that require long surgical time and much experience, and may not be suitable for every patient [17, 18]. Based on the clinical observation that incisions made in scarred areas bleed more than normal tissue, we hypothesized that trauma would have a positive effect on flap viability with the surgical delay effect, contrary to the general opinion, and thus determined to investigate the effects of different types of trauma and scars on flap viability in this study.

Posttraumatic hypoxia initiates capillary proliferation by the release of angiogenic factors, such as VEGF [6, 19]. Intercapillary anastomoses around the scar are created during the remodeling period, starting on the 7th day and provide sufficient blood flow to the tissue [20]. Edstrom et al. [9] showed that the survival rate of the random pattern flaps removed on the 42nd day from the scarred pedicle were similar to the rate observed in the control group, but when flaps were removed on the 14th, 21st, and 28th day, the survival rate of the flaps was much lower than the control group. Therefore, we chose a delay of 45 days after the trauma procedure before removing the flaps. Theile et al. [21] determined the viability rate of flaps with axial pattern planned from scarred tissue to be 98%. In contrast to these results, there are studies showing that the use of flaps removed from a scarred abdomen in breast reconstruction increases the risk of necrosis [10, 11].

The flaps created with a scarred pedicle (Group 2) tended to have a better survival rate than the flaps in the control group, but this was not significant. Microangiographic examination showed that the capillary density increased around the scar in Group 2 and contrast material passed through the scar and stained the distal vessels. However, high vascular density alone is not sufficient for adequate nutrition of the tissue and stabilization of the vascular network is required [20]. Theile et al. [21], when investigating the process of neoangiogenesis, showed that the highest flap survival rate was not observed on the 10th day, when the vessel formation was the highest, but continued to increase over time. Thus, not only are new vessels necessary for flap survival but there is also a requirement for vascular stabilization, which continues even after the peak of neoangiogenesis has occurred. Our findings are in agreement with these earlier studies and confirm that a flap can be safely planned over a scarred pedicle when there is sufficient time between the scarring and the raising of the flap. Based on the evidence of previous studies [9, 21], we hypothesized that a delay of 45 days from first procedure trauma would be sufficient for vascular stabilization.

The inflammatory process that occurs after trauma results in the opening of normally closed choke vessels and also increases the number of vessels present [22–24]. This process contributes to the delay phenomenon, which is the most reliable method known to increase flap survival [7]. In a study where preconditioning was performed as a result of microneedling with a dermaroller, it was found that the viability in the group which underwent microneedling was greater than that observed in the control group and similar to the viability found in the surgical delay group [25]. In our study, multiple traumas applied to the flap body (Group 3) were performed with full-thickness traumas on the whole body with an 18 G pink thick injector needle. In our study, the trauma applied to the flap with an 18 G injector needle was greater than the microneedling preconditioning. Group 3 had the highest flap survival rate among the experimental groups and this was similar to the control group. In addition, Group 3 tended to have the greatest number of vessels in the critical zone, although this was not significantly different from the other three groups. We suggest that trauma extending to deep tissues may have increased tissue hypoxia, made the inflammatory process more intense and consequently increased the neo-angiogenic process similar to the preconditioning used by Ünverdi and Çoruh [25], but in our study, this effect was more intense. Simultaneous initiation of this process in different parts of the tissue due to multiple scars is additional evidence for this hypothesis. On microangiographic examination this group also had the most extensive vascular network, even greater than in the control group and the distribution of contrast material throughout the flap was greatest, providing yet more evidence in support of some unidentified characteristic of the deeper tissue trauma resulting in better and more stable posttraumatic neoangiogenesis. Thus, it appears that scarring of the pedicle is no hindrance to the viability of a planned flap.

In the literature, there is no experimental study that has investigated the effect of scarring in the midline of the flap. However, there are some clinical studies that have recommended avoidance of the use of abdominal tissue with a midline scar as a flap in breast reconstruction [10, 11]. In these studies, when deep inferior epigastric perforator flap and transverse rectus abdominus myocutaneous flap were used, it was reported that the risk of necrosis in the distal region of the flaps was increased when there was a previous midline scar. In Group 4, where the scar was created at the midline of the planned flap, the survival rate was statistically lower than the control group and other groups (p < 0.05). In addition, in Group 4, no capillary network was found in any of the subjects using microangiographic imaging in the area of the flap distal to the scar. There may be a number of mechanisms to explain this finding. Firstly, if there is insufficient hypoxia to adequately stimulate neovascularization, due to the preexisting rich capillary network around the scar line of the dorsal flap, then no new capillaries would be formed in this region after the flap is created. This may be an effect of the model tissue used that is a characteristic of rat dorsal skin vascular anatomy. Secondly, when the flap is elevated, the pressure of the blood flow coming from the pedicle decreases as it moves distally, and is insufficient to reach the regions distal to the scar tissue. Thus, the scar tissue may act as a barrier to blood flow. However, extending the time between trauma and harvesting the flap can eliminate this problem. Tuncel et al. [26] argued that arteries redevelop in an axial pattern in the scar tissue and that arteries are more likely than veins to form in scars, so that necrosis is mostly due to venous insufficiency in flaps elevated on scarred pedicles. In another experimental study performed in the rat abdominal area, a flap fed by a scarred pedicle increased the viability of the flap with an arterial or venous support provided from the opposite side without scar and that there was no difference if the supporting vessel was a vein or an artery [27]. In Group 4, the poor blood supply from the healthy side to the scar may be the main cause of reduced viability. Interestingly, the number of vessels in the critical zone in Group 4 was higher than the control group. This may be because the vascular connections that cannot pass into the distal area of the scar are concentrated proximally. Similar to previous clinical studies [10–12], our results show that the risk of distal necrosis increased in cases where the preexisting scar was present in the midline of the planned flap.

Our study appears to show that, when planning flaps, the location of the scar is very important. Evidence for this emerged when comparing Group 2 and Group 4. These two groups underwent similar procedures but the location of the scars was different. In Group 2, flap survival rates were significantly higher than in Group 4. This may be explained by the scar acting as a barrier for the blood stream. When the scar was located on the pedicle side (Group 2), blood flow pressure was sufficient to pass the scar because the blood was coming from healthy rat dorsal skin. However, when the scar was located in the middle of the flap (Group 4), the blood flow pressure was low, the blood supply was coming from the body of the flap; thus, the pressure of the blood flow was lower than in healthy rat dorsal skin. Thus, the pressure was insufficient for the blood supply to pass the barrier formed by the scar.

When PMNL infiltration, edema, inflammation, increased fibroblast activity, fibrosis, and neovascularization were evaluated, there was no statistically significant difference between the groups. PMNL infiltration and edema are signs of early phases of inflammation, while increased fibroblast activity and fibrosis are late phase signs. Previous studies have shown the association of these markers of inflammation with poorer flap viability. In the experimental groups the histopathological scores assigned to these markers were similar across the groups. There may be two reasons for this; the first is the similar survival rates observed in all groups and the second is the subjective nature of the assessment.

There is an important limitation of our study. It should be noted that wound healing in rat skin is faster than in humans, which limits the clinical applicability of the study. Thus, there may be a place for further experiments in organisms with skin anatomy more similar to humans, such as the pig. In addition, the experimental delay after the trauma procedure was 45 days. It may be that different results would have been observed if this delay was extended further and, as rats heal more rapidly than humans, this delay period would need to be determined in clinical studies in human patients undergoing normal reconstructive surgeries.

## 5. Conclusion

There are many studies in the literature evaluating the use of scar tissue as flaps. The evaluation of the usability of tissues when the scar is in the middle of the planned flap and which has been traumatized throughout the entire body of the flap tissue distinguishes our study from others. The results of our study showed that tissues with a previously cut pedicle or full-thickness trauma along the body may be used as flaps in the long term in our model. However, the use of tissues in which the middle of the flap contains a scar that will be included in the planned flap should be avoided. It must be noted that the human body is very different from that of the rat, so that the use of scar tissue in flap surgery requires more experimental and clinical evidence before this practice could be safely adopted in surgery on humans.

## Figures and Tables

**Table 1:** Study schedule.

	Day - 0	Day - 7	Day - 45	Day - 52
Group 1 (C)	Flap elevation	Flap survival measurement-microangiography-biopsy		
Group 2 (PI)	Pedicle incision		Flap elevation	Flap survival measurement-microangiography-biopsy
Group 3 (PREC)	Preconditioning with flap body traumatization		Flap elevation	Flap survival measurement-microangiography-biopsy
Group 4 (MI)	Middle incision		Flap elevation	Flap survival measurement-microangiography-biopsy

**Table 2:** Outline mean percentage of flap survival in the groups.

	Group 1 (C) mean ± SD median (25–75)	Group 2 (PI) mean ± SD median (25–75)	Group 3 (PREC) mean ± SD median (25–75)	Group 4 (MI) mean ± SD median (25–75)	*p*
Total flap area (mm^2^)	2237.16 ± 75.89	2202.73 ± 100.42	2282.57 ± 102.60	2237.36 ± 143.53	0.372
Flap survival area (mm^2^), %	%66.78 ± 7.00^a^	%68.05 ± 7.58^b^	%68.50 ± 6.16^c^	%60.01 ± 4.72^a^^b^^c^	0.011

a significant difference between group 1 and group 4 (p: 0.043)

b significant difference between group 2 and group 4 (p: 0.028)

c significant difference between group 3 and group 4 (p: 0.016)

SD: standart deviation

C: control; PI: pedicle incision; PREC: preconditioning; MI: middle incision

**Table 3:** Number of vessels in healthy skin 1 cm proximal to the necrotic border.

	Group 1 (C) mean ± SD median (25–75)	Group 2 (PI) mean ± SD median (25–75)	Group 3 (PREC) mean ± SD median (25–75)	Group 4 (MI) mean ± SD median (25–75)	*p*
Number of vessels	27.92 ± 14.81	27.91 ± 11.80	36.67 ± 8.13	38.00 ± 8.87	0.058

SD: standard deviation

C: control; PI: pedicle incision; PREC: preconditioning; MI: middle incision

**Table 4:** Results of the histopathological examination.

	Group 1 (C) mean ± SD median (25–75)	Group 2 (I) mean ± SD median (25–75)	Group 3 (PREC) mean ± SD median (25–75)	Group 4 (MI) mean ± SD median (25–75)	*p* ^*^
PMNL	0.83 ± 0.71	1.36 ± 1.02	0.83 ± 0.38	0.45 ± 0.68	0.081
Fibrosis	1.83 ± 0.93	1.72 ± 0.78	1.41 ± 0.51	1.18 ± 0.60	0.140
Edema	1.66 ± 1.37	1.81 ± 0.60	2.33 ± 0.77	2.00 ± 0.77	0.241
Inflammation	2.00 ± 0.73	2.45 ± 0.93	2.50 ± 0.52	2.18 ± 0.75	0.342
Fibroblast activation	1.83 ± 1.11	2.36 ± 0.67	2.00 ± 0.60	1.90 ± 0.70	0.313
Neovascularization	2.08 ± 1.31	2.18 ± 1.16	3.08 ± 0.79	3.09 ± 0.83	0.061

SD: standard deviation, (*p > 0.05)

C: control; PI: pedicle incision; PREC: preconditioning; MI: middle incision; PMNL: polimorphonuclear leucsyt).
